# Supplementary material for: Spatiotemporal dynamics of locomotor decisions in Drosophila melanogaster
Source: bioRxiv. 2024 Sep 5:2024.09.04.611038. Preprint. [Version 1] doi: 10.1101/2024.09.04.611038 (PMC11398310; doi:10.1101/2024.09.04.611038)
Supplement: Supplement 1 [file NIHPP2024.09.04.611038v1-supplement-1.pdf]

bioRxiv preprint doi: https://doi.org/10.1101/2024.09.04.611038; this version posted September 5, 2024. The copyright holder for this preprint (which was not certified by peer review) is the author/funder, who has granted bioRxiv a license to display the preprint in perpetuity. It is made available under aCC-BY 4.0 International license.

Supplementary Information

Simulated agent-based fly models (ABM)

To investigate whether we could approximate fly decision-making in a Y-maze using only simple movement rules, we employed an agent-based modeling (ABM) approach. In this modeling framework, a simulated fly is treated as a circle of a constant radius, r_0, traversing the maze and constrained by the surrounding walls. Its initial position is drawn from a distribution of valid positions, in which the entire circle lies within the maze. For each of the remaining steps, the position is updated based on a distribution of heading angles, speed, and distance to the surrounding walls of the maze. The simulated agent makes repeated turn-decisions, until the predetermined simulation duration is reached. We used scaled parameters that match the body size and speed of a WT fly in the short maze, as well as the framerate of the assay camera. We examined the behavior of different agents by implementing different position update rules on the distribution of heading angles and simulated multiple flies in each.

In its simplest form, which we use here as a baseline, a simulated agent in the above ABM framework follows a constrained Brownian motion. Specifically, the heading angle in each frame, which was matched to the sampling rate of the preceding behavioral assays, is drawn from a Uniform distribution (between 0 and 359) and the resulting direction is only constrained such that the circle is entirely confined by the maze walls. Angles are defined in such a way that 0 and 360 denote forward facing, and 180 denotes backwards. To further understand the motion choice dynamics exhibited by real flies, we extended the ABM by: (1) introducing heading angle choices depending on past angles correlated rather than random heading angles and, (2) incorporating a tunable parameter, WF, governing wall-following tendencies. In summary, for every frame f, simulated flies randomly pull a heading angle α from a Gaussian distribution of mean μ = 0, and variance $\boldsymbol{\sigma}$. The resulting heading angle is then used to update the last recorded heading direction. Next, simulated fly's position is attempted to be updated based on the resulting heading direction and the fly's speed s, which approximated real flies' speed in mm/frame in the behavioral assays. If the proposed position would result in any part of the fly body circle to be inside the environment walls or outside of bounds, the closest valid position is instead chosen. Closest valid position is defined as the coordinate that (a) would result in the smallest current heading angle change and (b) would result in no part of the fly body circle to be inside a wall or outside of bounds. Wall attraction was set to occur to a degree given by WF whenever the distance, $d(t)$, between the current position (centroid) of the simulated fly and the closest wall coordinate falls below a predefined fixed parameter r_w ($r_w > r_0$; see Materials and Methods). Thus, for a given value of WF the agent updates its heading angle only based on (1), unless $d(t) < r_w$, in which case the chosen heading angle is updated by the WF-weighted mean of the angle towards the closest wall and the current heading angle, as described in (2).

We simulated a first set of 3 experiments of increasing complexity using a combination of the simple rules described. In the first experiment, we simulated flies that followed Brownian motion. Every frame, heading angle was randomly chosen to be between 0 and 359, and heading angle was not updated based on wall proximity. In the second experiment, simulated flies followed momentum-retaining movement rules as described above, but did not update their heading angle

bioRxiv preprint doi: https://doi.org/10.1101/2024.09.04.611038; this version posted September 5, 2024. The copyright holder for this preprint (which was not certified by peer review) is the author/funder, who has granted bioRxiv a license to display the preprint in perpetuity. It is made available under aCC-BY 4.0 International license.

depending on wall proximity. In the third experiment, we simulated flies using movement rules and wall proximity-based heading angle updates as described above. Additionally, to investigate the effect of wall-following on simulated flies' motion and decision-making predictability readout, we performed a parameter sweep on the wall proximity-dependent angle weight averaging parameter WF. Over a second set of 5 additional experiments, we systematically increased WF from 0, which equals the previously described second experiment, to 0.1, which equals the previously described third experiment. We simulated 100 flies per experiment, chosen to approximate behavioral assay sample sizes. Flies were simulated for 108000 steps, mirroring a sampling rate of 30hz over a 1h experiment. To alleviate Brownian-motion dependent relatively low number of scorable trials, flies in the first experiment were instead simulated for 540000 steps. Resulting datasets were analyzed in the same fashion as datasets originating from behavioral assays. We analyzed movement only after reaching the lowest point in the cul-de-sac ($y' = 0$), as Brownian agents repeatedly traversed the same y-bin, rendering the distinction between $-y'_{Tr,k}$ and $y'_{Tr,k}$ meaningless.

First, we found that TPI curves of Brownian agents appeared very different from real flies (Fig. S13A). Given the large degree of noise in this ABM version, the Brownian agent displays numerous downward/upwards traversals between the bottom arm and the intersection in a single trial before eventually entering either the left or right arm (Fig. S14). This is in contrast to real flies that typically make single downward and upward traverses in each trial. Critically, with the last midline-crossing and, hence, the increase in predictability occurring only very close to the intersection, the behavior of Brownian agents substantially deviates from the behavior of real flies (c.f., Figs. S13A & 4A). Moreover, the number of midline-crossings during upward motion made by these simulated agents is many folds larger than that of flies (c.f., Figs. S6C, right & S13E, top right). Thus, the Brownian ABM does not replicate the core determinants of the motion choice dynamics observed in real flies.

Second, the behavior of $WF = 0$ and Brownian agents differ in two main aspects. The average number of midline-crossings after the cul-de-sac is of a similar magnitude to those of real flies (c.f., Figs S13E, left, dark red & S6C, right). In contrast to Brownian agents and real flies, the predictability of no wall-following agents decreases *below* zero during their upward motion through the arm (Fig. S13A, top, dark red), capturing their tendency to follow the wall *incongruent* with their eventual turn direction (Fig. S15D). Increasing WF above zero relaxes this turn-incongruent wall-following effect (e.g., see $WF = 0.01$, orange curve in Fig. S13A, top) whereas an opposite, turn-*congruent* wall-following effect is displayed by agents with sufficiently large WF ($WF \geq 0.05$ in Fig. S13A). As WF is further increased, the central tendency of the distribution of last midline-crossings shifts towards lower y values (Fig. S13A, bottom), reflecting an earlier onset of wall-following (Fig. S15D) and, consequently, an earlier increase in predictability above zero (Fig. S13A, top).

Third, considering global lateral tendencies and, as could be expected from the earlier onset of their effective wall-following (Fig. S15D), agents with larger WF not only display TPI curves above those with lower WF (Fig. S13A, top) but also exhibit increased MAD scores during upward motion (Fig. S13B). This is in line with differences between WT flies and flies of most mutant lines (See: Figs. 4A, top & 4B, top). However, in contrast to real flies, P_{Even}, the local tendency to make even

bioRxiv preprint doi: https://doi.org/10.1101/2024.09.04.611038; this version posted September 5, 2024. The copyright holder for this preprint (which was not certified by peer review) is the author/funder, who has granted bioRxiv a license to display the preprint in perpetuity. It is made available under aCC-BY 4.0 International license.

amounts of midline-crossings after the cul-de-sac, deviates from chance only for agent with large WF (Fig. S13C-D: $WF = 0.09$ & $WF = 0.1$, dark blue & purple, respectively). That is, because, with their (effective) later onset of wall-following, the horizontal location *within* the cul-de-sac for agents with lower WF is almost uninformative of their eventual turn-direction (see $WF = 0.05, 0.07$ in Figs. S15D & S13A). Conversely, for agents with larger WF ($WF = 0.09, 0.1$), the horizontal location within the cul-de-sac is informative of the eventual turn direction. Naively, this could suggest that the way these agents make turns is somehow similar to the turn-decision process of real flies. However, upon closer examination, it becomes clear that this is not the case. This is because, in contrast to the behavior of real flies, for which predictability typically starts deviating from zero *within* the cul-de-sac (Figs 4A & S6A, middle) and is sustained by means of local lateral tendencies, a change in predictability within the cul-de-sac for all ABM versions is either absent or reflects the magnitude of predictability already when entering the cul-de-sac ($WF \leq 0.07$ and $WF \geq 0.09$, respectively, in Figs. S13A, top & S15A, center). Indeed, in more than 90% of the trials, $WF = 0.09$ agents make a single midline-crossing within the cul-de-sac (Fig. S13E, right), which expresses their enduring wall-following tendency even before the cul-de-sac. This tendency is even further pronounced in $WF = 0.1$ agents, which wall-follow the surrounding wall over almost the entire maze (Figs. S13A, top, & S13E). Thus, the midline-crossings made by ABM agents typically reflect their movement *before* committing to the wall to which they persist following ($WF \leq 0.07$) or simply the endurance of wall-following that started before the cul-de-sac by means of a single midline-crossing ($WF \geq 0.09$). Post wall-commitment, movement of ABM agents is almost deterministic: they typically make either zero or a single midline-crossing and therefore, a local lateral tendency metric of the parity in the amount of midline-crossing after this location adds no information about the eventual turn-direction.

Thus, the wall-following agents introduced by the ABM framework cannot replicate the capacity of real flies to sustain their probabilistic motion signatures by means of local tendencies. As TPI is tightly linked with the tendency to make even midline-crossings throughout the remainder of the trajectory, we conclude that TPI curves of real flies and simulated ABM agents reflect very different decision processes. These results further emphasize that flies do not solely rely on wall-following. That is, there must exist decision processes beyond simple movement rules that flies rely on when making decisions across space and time.

Quiver plots

To create the average quiver plots shown in Fig. 4D, we computed two primary variables for each fly in each genetic line under four conditions: left turns (blue), right turns (red), and motion towards ($y < 0$) and away $y > 0$) from the cul-de-sac edge. These variables were the spatial density and the average movement direction. In what follows, we describe these computations for an individual fly and finally, how each average quiver is computed across flies.

The cul-de-sac was divided into a 28-by-22 2D grid with bins defined by $X = \{-0.2268, \ldots, 0, 0.0162, \ldots, 0.2268\}$ and $Y = \{0, 0.0162, \ldots, 0.3564\}$ (or $Y = \{-0.3564, -0.0162, \ldots, 0\}$ for $y < 0$). For each frame f of a fly's trajectory within the cul-de-sac, we determined the bin (i, j) corresponding to the coordinates (x_f, y_f) and incremented the count for that bin: $counts(i, j) = \sum_{f=1}^{F} \delta(x_f \in bin_i, y_f \in bin_j)$, where δ is the indicator function that equals 1 if (x_f, y_f) is within bin

bioRxiv preprint doi: https://doi.org/10.1101/2024.09.04.611038; this version posted September 5, 2024. The copyright holder for this preprint (which was not certified by peer review) is the author/funder, who has granted bioRxiv a license to display the preprint in perpetuity. It is made available under aCC-BY 4.0 International license.

(i, j) and 0 otherwise, and F is the total number of frames across all trials. To compute the average movement direction for each frame f, we calculated the velocity vector $v_f = (x_{f+1} - x_f, y_{f+1} - y_f)$ and computed the corresponding angle $\theta_f = arctan2(y_{f+1} - y_f, x_{f+1} - x_f)$. For each bin (i, j), we accumulated these angles and their corresponding magnitudes $|v_f|$. The magnitude-weighted average angle $\theta_{i,j}$ for bin (i, j) was computed by converting each angle θ_f to its complex representation $e^{i\theta_f}$ and computing the weighted sum of these complex representations: $\theta_{i.j} = arg\left\{\frac{\sum_{f=1}^{F} v_f|\cdot e^{i\theta_f}|}{\sum_{f=1}^{F}|v_f|}\right\}$.

To compute the average quiver over multiple flies, we combined the corresponding $counts_{i,j}$ and $\theta_{i.j}$ from individual flies. The $counts$ of each fly were normalized to represent the relative frequency of visits to each bin, $freqs_{i,j,n}$, where n indexes the flies. The average direction angles $(\theta_{i.j,n})$ for each fly were converted to their complex representation, $e^{i\theta_{i.j,n}}$. For each bin, we computed a frequency-based weighted average of the angles across all flies: $< \theta_{i.j} >_{flies} = arg\left\{\frac{\sum_{n=1}^{N} freqs_{i,j,n}\cdot e^{i\Theta_{i,j,n}}}{\sum_{n=1}^{N} freqs_{i,j,n}}\right\}$. The relative frequency of visits to each bin was averaged over all flies to get the average frequency with which each bin was visited: $< freqs_{i.j} >_{flies} = \frac{1}{N}\sum_{n=1}^{N} freqs_{i,j,n}$. The computed average direction ($< \theta_{i.j} >_{flies}$) and frequency ($< freqs_{i.j} >_{flies}$) for each bin were used to plot the average quiver. The direction of each vector in the average quiver plot corresponds to the average direction of movement, while the magnitude of each vector represents the relative frequency of visits to that bin.

Heat maps

In addition to the quiver plots, we computed average heat maps over flies (or simulated agents) to visualize the spatial distribution of their locations during motion away from the cul-de-sac $y > 0$. In what follows, we describe the computation for an individual fly and finally, how the average heatmap is computed across flies.

The maze was divided into a 2D grid of bins defined by $X = \{-0.1944, \ldots, 0, 0.0324, \ldots, 0.1944\}$ and $Y = \{0, 0.0324, \ldots, 1.4580, 1.4904\}$. The spatial probability for each bin (i, j) was defined by: $binsProb_{i,j} = \frac{1}{F}\cdot count(x_f \in bin_i, y_f \in bin_j)$, where f is a frame in the trajectory data of a fly, F is the total number of frames for that fly that lay in the 2D grid and $count$ is the number of frames where (x_f, y_f) falls within bin (i, j).

To compute the average heat map over multiple flies for each turn direction (left or right), the spatial probability ($binsProb$) for each fly was computed separately for left and right turns, and then normalized to represent the probability distribution across bins (Fig. 2B, top and center). For each bin (i, j), the average probability was computed across all flies: $< binsProb_{i,j} >_{flies} = \frac{1}{N}\cdot\sum_{n=1}^{N} binsProb_{i,j,n}$. To visualize the differences between left and right turns, we also computed $\Delta binsProb_{i,j} =< binsProb_{i,j,right} >_{flies} -< binsProb_{i,j,left} >_{flies}$ (Figs. 2B, bottom, & S15D).

Zero-midline-crossings comparison between mutants and WT

bioRxiv preprint doi: https://doi.org/10.1101/2024.09.04.611038; this version posted September 5, 2024. The copyright holder for this preprint (which was not certified by peer review) is the author/funder, who has granted bioRxiv a license to display the preprint in perpetuity. It is made available under aCC-BY 4.0 International license.

To assess the contribution of trials with zero midline-crossings to the sharper increase in predictability within the cul-de-sac for WT and mutant flies, we computed $TPI(yRange)$ after excluding trials in which there were zero midline-crossings after the cul-de-sac. While this exclusion involves an artificial debiasing of the parity tendencies of all flies and thus expected to result in reduced predictability (i.e., because excluding zero midline-crossing trials necessarily reduces the probability to make turns congruent with the horizontal location in the cul-de-sac towards chance level), we use it as a metric to estimate the departure in predictability between WT and mutant flies with respect to their horizontal location just before they leave the cul-de-sac. Figures S6D and S6A, bottom depict the average TPIs for positive midline-crossings and their corresponding estimates for the change in predictability within the cul-de-sac. While FoxP, NorpA, and NompC mutant lines all differed significantly in their cul-de-sac TPI values when considering all trials, a substantial effect persisted primarily in Dumb flies after zero-omission (Fig. S6A, top).

Simulated midline-crossings

To simulate the distributions of $\#crossings$ for mazes of different arm length we considered the midline-crossings made by flies in the long maze. Recall that $\#crossings$ is defined as the number of midline-crossings during upward motion away from the cul-de-sac (Fig S5A; see also Fig. 3D). Because flies in the long maze rarely make midline-crossing after leaving the intersection (0.011%, 4 crossings out of 35,759 post cul-de-sac midline-crossings made by flies in the long maze) then effectively, these midline-crossings typically occur in the interval between the upper edge of the cul-de-sac and the upper edge of the intersection (see Fig. S3B-C). Specifically, the length of this interval is $2d + k$ in the long maze, where $2d$ is the length of each arm in the long maze and k is the length of the intersection (between its bottom and top ends). The corresponding length of this interval in the short maze is $d + k$ and, for any maze with different arms' length, the interval can be similarly described as $rd + k$, where r is the arm-length ratio of any maze to the short maze. To consider the number of midline-crossings expected in mazes with different arm length ($r = \{.1, .33, .5, .66, .9, 1, 1.1, 1.33, 1.5, 1.66, 1.9, 2\}$) we computed, for each trial made by flies in the long maze, the number of (the first) midline-crossings occurring before the $rd + k$ interval is exceeded. Bootstrapping trials from the resulting trimmed $\#crossings$ for each r results in the expected distributions seen in Figure S5D. The resulting $\#crossings$ distribution for $r = 1$ replicates the observed $\#crossings$ distribution of flies in the short maze and hence the tendency to make even $\#crossings$ across the sample. This tendency is also expected from mazes with arm-length either larger ($r > 1$) or smaller than the short maze ($r < 1$).

We also simulated the $\#crossing$ distributions expected in the short and long mazes under the assumption that midline-crossings are given by draws from an inter-crossing-interval distribution. For this purpose, we computed for each fly and each trial in the long maze the inter-crossing intervals. For each trial, the y-locations of all post cul-de-sac midline-crossings are given by $y_j|cross$, where $1 \leq j \leq k$ and k is the number of post cul-de-sac crossings in that trial. Defining $y_0|cross$ and $y_{k+1}|cross$ as the y-values denoting the y-locations of the cul-de-sac upper edge and the intersection upper edge (respectively; similar across all flies and trials), the inter-crossing-distances in that interval, measured in distance only on the y-axis, is given by $\Delta y_j = y_j|cross - y_{j-1}|cross$, where $1 \leq j \leq k + 1$. To consider the expected distribution of $\#crossings$ under the

bioRxiv preprint doi: https://doi.org/10.1101/2024.09.04.611038; this version posted September 5, 2024. The copyright holder for this preprint (which was not certified by peer review) is the author/funder, who has granted bioRxiv a license to display the preprint in perpetuity. It is made available under aCC-BY 4.0 International license.

above assumption in either the short or long mazes, we sampled $\#trials(f)$ trials from each fly in the ling maze, where $\#trials(f)$ is the number of trials made by fly f in the long maze. For each simulated trial, we sampled Δy values from all Δy across all trials made by this fly. To avoid over-sampling of trials with numerous $\#crossings$ (and hence small Δy's) the sampling weight (probability) ${w_j}^t$ of each ${\Delta y_j}^t$, an inter-crossing-distance of the fly in trial t, was set to $[(k+1) \cdot \#trials(f)]^{-1}$. For each trial, Δys were sampled until $\sum_{s=1}^{S} \Delta y_s$, the accumulated Δy's in the simulated trial reached the interval between the upper edge of the cul-de-sac and the upper edge of the intersection (long maze: $2d+k$, short maze: $2d+k$). The resulting number of midline-crossings in the simulated trials is given by the $S-1$, where S is the number of sampled Δy's when the interval is crossed for the first time. Simulating $\#trials(f)$ for each fly f for each of the flies resulted in one simulated distribution of $\#crossings$ under the above assumption of draws from a distribution of inter-crossing-distances. Repeating this procedure 1000 times results in the expected distributions in Fig. S5B. These simulations did not replicate the observed distributions of $\#crossings$ in short and long mazes (Fig. S5B). In an attempt to test whether this relates to some differences between midline-crossing made within the maze's bottom arm and those made within the intersection, we repeated the above process by computing and simulating only Δy and $\sum_{s=1}^{S} \Delta y_s$ occurring within the arm (intervals for long and short mazes: $2d$ and d, resp.). The simulated $\#crossings$ in a trial of that revised procedure was given by the sum of $S-1$, the number of sampled Δy's when the revised interval ($2d$ or d) was crossed for the first time and the observed number of midline-crossings occurring within the intersection in that trial. This revised procedure failed to replicate the observed distributions of $\#crossings$ in short and long mazes (Fig. S5C).

bioRxiv preprint doi: https://doi.org/10.1101/2024.09.04.611038; this version posted September 5, 2024. The copyright holder for this preprint (which was not certified by peer review) is the author/funder, who has granted bioRxiv a license to display the preprint in perpetuity. It is made available under aCC-BY 4.0 International license.

Table S1. Sample sizes and exclusion criteria.

Sample Size	*WT short*	*WT long*	*FoxP*	*NorpA*	*Dumb*	*NompC*	*Humans*
post visual inspection	*99*	*96*	*288*	*258*	*136*	*63*	*35*
post #trials threshold	*67*	*56*	*243*	*249*	*92*	*56*	*30*
post tracking error rejection (final sample size)	*55*	*50*	*243*	*247*	*90*	*54*	*30*

bioRxiv preprint doi: https://doi.org/10.1101/2024.09.04.611038; this version posted September 5, 2024. The copyright holder for this preprint (which was not certified by peer review) is the author/funder, who has granted bioRxiv a license to display the preprint in perpetuity. It is made available under aCC-BY 4.0 International license.

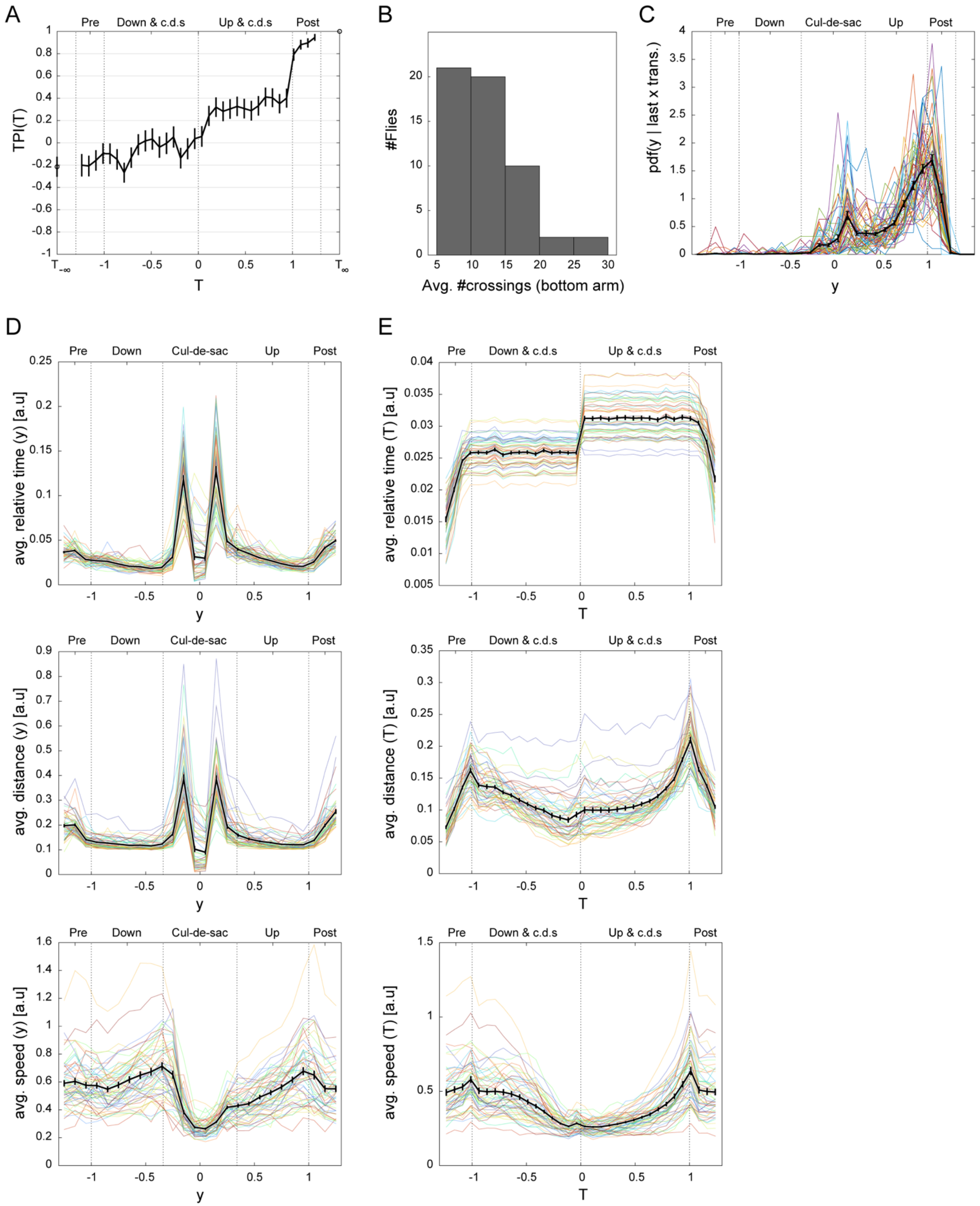

bioRxiv preprint doi: https://doi.org/10.1101/2024.09.04.611038; this version posted September 5, 2024. The copyright holder for this preprint (which was not certified by peer review) is the author/funder, who has granted bioRxiv a license to display the preprint in perpetuity. It is made available under aCC-BY 4.0 International license.

Figure S1. Flies in the short Y-Maze. (A) Turn Predictiveness Index (TPI) in the temporal domain for the fly in Fig. 1B-D. $TPI(tRange)$ measures, for a given $tRange$, how predictable the average x location of a fly is with respect to its forthcoming turn direction (see Materials and Methods). Error bars denote Standard Error (SE). (B) Average midline-crossing histogram. The histogram denotes the trial-average amount of midline-crossings (passes through $x = 0$) made by each fly ($n = 55$) within the bottom arm in each trial ($-1 < y < 1$). (C) Average PDF of last midline-crossings (LMC). LMC is defined as the last y-location within a given trial in which the x-location changed polarity (i.e., last crossing of the horizontal midline). The average $PDF(y \mid LMC)$ curve (Black) over the entire sample ($n = 55$; WT) is overlaid on the $PDF(y \mid LMC)$ curves of individual flies. Error bars denote Standard Error of the Mean (SEM). (D) Average motion kinematics of flies across the maze, depicting average relative time (top), distance (center) and speed (bottom), computed for the same y-bins in Fig. 1F. The average curves across flies (Black; $n = 55$) is overlaid on the trials-average curves of individual flies. (E) As in D for the *relative* time-bins in Fig. 1G.

bioRxiv preprint doi: https://doi.org/10.1101/2024.09.04.611038; this version posted September 5, 2024. The copyright holder for this preprint (which was not certified by peer review) is the author/funder, who has granted bioRxiv a license to display the preprint in perpetuity. It is made available under aCC-BY 4.0 International license.

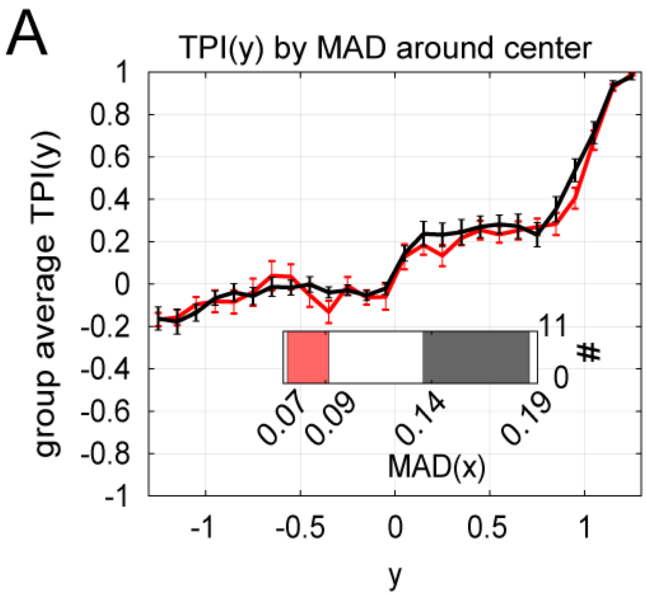

Figure S2. Average TPIs for flies in the short maze with lowest (red) and highest (nlack) $MAD(x|-1 < y < -0.34)$ ($n = 11$ in each group; WT). Inset: distribution of $MAD(x|-1 < y < -0.34)$ values over the sample. The $MAD(x|-1 < y < -0.34)$ value of each fly computes the median absolute deviation from the horizontal midline for *downward* motion from the upper edge of the bottom arm until entering the cul-de-sac (c.f., Figs. S2 & 2E).

bioRxiv preprint doi: https://doi.org/10.1101/2024.09.04.611038; this version posted September 5, 2024. The copyright holder for this preprint (which was not certified by peer review) is the author/funder, who has granted bioRxiv a license to display the preprint in perpetuity. It is made available under aCC-BY 4.0 International license.

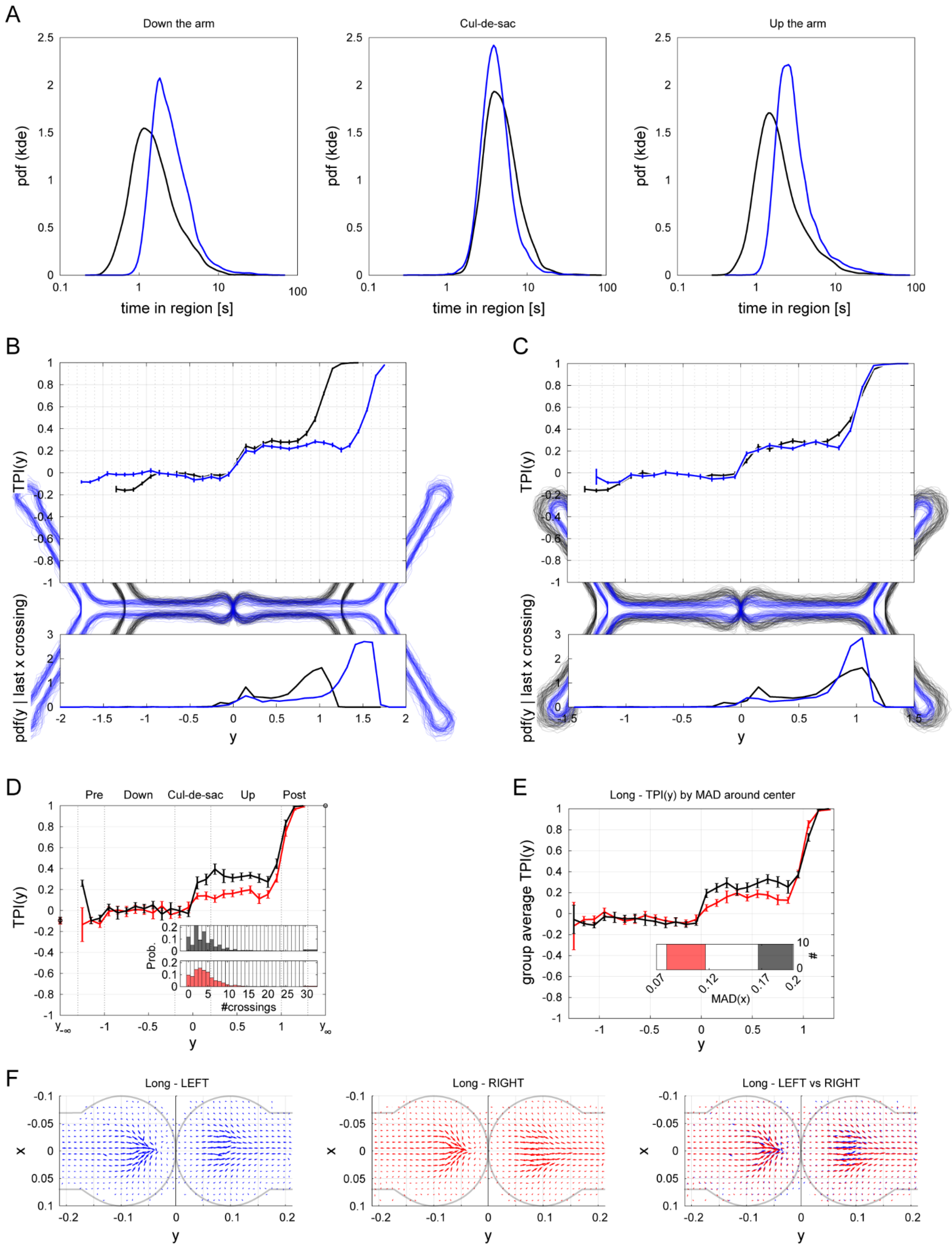

bioRxiv preprint doi: https://doi.org/10.1101/2024.09.04.611038; this version posted September 5, 2024. The copyright holder for this preprint (which was not certified by peer review) is the author/funder, who has granted bioRxiv a license to display the preprint in perpetuity. It is made available under aCC-BY 4.0 International license.

Figure S3. Long vs short mazes in the spatial domain. (A) Probability density functions (PDFs) of the absolute (to not confuse with relative) time spent walking down the arm (left), within the cul-de-sac (middle) and walking up the arm (right) for WT flies in the short (black; $n = 55$) and long (blue; $n = 50$) mazes. Each curve depicts a Kernel smoothing function estimate over log10 of the time spent in all trials across the sample. (B) Average TPI curves (top) and corresponding PDF of last midline-crossings (bottom) in absolute-size representation. All bins are of equal width and represent equal size segmentations of the raw plane (background images). (C) As in B, for relative representation of the plane, with $y = \pm 1$ defining the upper edge of the bottom arm (bottom edge of the intersection). (D) Average TPIs for flies in the long maze with lowest (red) and highest (black) P_{Even}values (calculated as in Fig. 3D, Top, blue; n=10 in each group). Inset: Probability mass functions of $\#crossings$ during upward motion away from the cul-de-sac in each group (color coded as in the main panel; $\#crossings$ def. as in Fig. 3D, bottom). (E) As in D, for global lateral tendencies in the long maze: Average TPIs for flies with lowest (red) and highest (black) MAD scores ($n = 11$ in each group). Inset: distribution of (MAD scores during upward movement through the arm) over the sample. (F) Quiver plots depicting average motion within the cul-de-sac across flies in the long maze. Y-coordinates in D-F are standardized as in C. For long vs short: c.f., Figs. S3D & 2D, S3E & 2E, S3F & 2A.

bioRxiv preprint doi: https://doi.org/10.1101/2024.09.04.611038; this version posted September 5, 2024. The copyright holder for this preprint (which was not certified by peer review) is the author/funder, who has granted bioRxiv a license to display the preprint in perpetuity. It is made available under aCC-BY 4.0 International license.

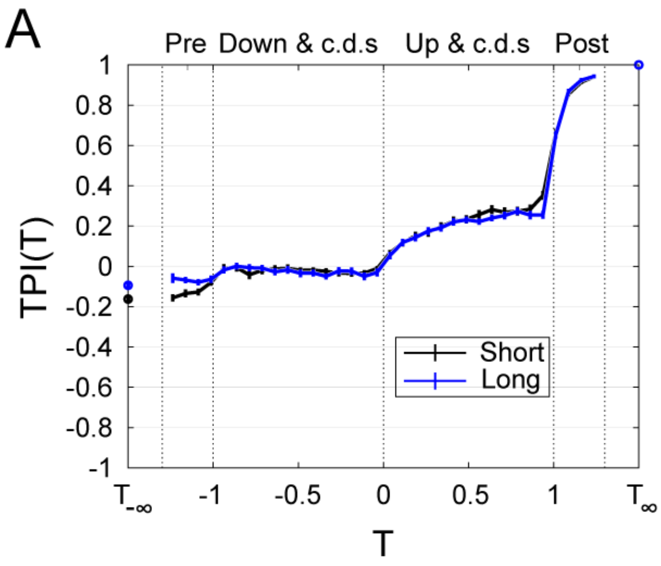

Figure S4. Long (blue; $n = 50$) vs short (black; $n = 55$) average TPI curves in the temporal domain. Error bars denote Standard Error of the Mean (SEM).

bioRxiv preprint doi: https://doi.org/10.1101/2024.09.04.611038; this version posted September 5, 2024. The copyright holder for this preprint (which was not certified by peer review) is the author/funder, who has granted bioRxiv a license to display the preprint in perpetuity. It is made available under aCC-BY 4.0 International license.

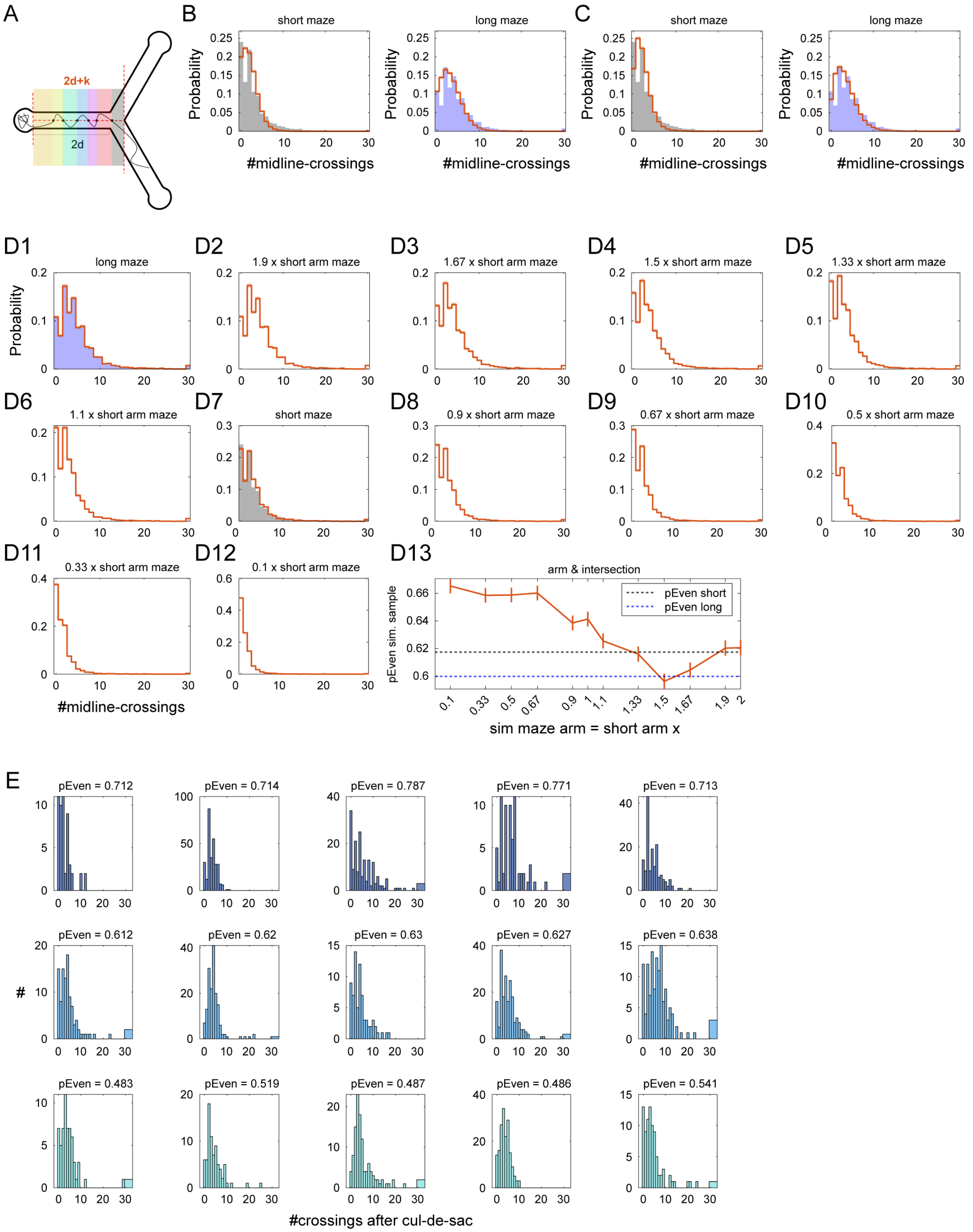

Figure S5. Post cul-de-sac midline-crossings in the long maze and corresponding crossing simulations. (A) Illustration of post cul-de-sac midline-crossings in a trial. After leaving the cul-de-

bioRxiv preprint doi: https://doi.org/10.1101/2024.09.04.611038; this version posted September 5, 2024. The copyright holder for this preprint (which was not certified by peer review) is the author/funder, who has granted bioRxiv a license to display the preprint in perpetuity. It is made available under aCC-BY 4.0 International license.

sac, the illustrated trajectory passes through the horizontal midline 6 times and, thus, $\#crossings = 6$. The same definition for $\#crossings$ is used in Figs. 2C-D, 3D, 4C, S3D, S6C, right, S6D, S9, S10, S11B, and S13C-E, left. Because flies in the long maze almost never make midline-crossing after the intersection (0.011%, 4 crossings out of the 35,759 post cul-de-sac midline-crossings in Fig. 3D, bottom, blue) then, effectively, post cul-de-sac midline-crossings are constrained within a $2d + k$ interval between the upper edge of the cul-de-sac and the upper edge of the intersection, where $2d$ is the length of the arm in the long maze (d in the short maze, $r \cdot d$ in any maze with arms' length r times the short maze) and k is the length of the intersection (similar across mazes). (B) Simulating inter-crossing-y-intervals does not replicate the observed $\#crossings$ distributions in the short (left) and long (right) mazes. Filled histogram: observed $\#crossings$ pdf (as in Fig. 3D, bottom). Orange line and shaded area: simulated $\#crossings$ pdf, averaged over 1000 simulations and simulations' std, respectively. In each simulation ($n_{sims} = 1{,}000$) and each fly in the long maze ($n = 50$) we simulated the number of crossings in each trial by sampling from the fly's observed post cul-de-sac inter-crossing-y-distances across all trials (see Supporting Information). The simulated number of post cul-de-sac crossings in each trial was determined when the accumulated inter-crossing-y-distances in a simulated trial attained $d + k$ or $2d + k$ for the short (left) and long (right) maze simulations, respectively. (C) As in B, simulating the number of post cul-de-sac crossings in the short (left) and long (right) mazes. But, using the inter-crossing-y-intervals that occur only *within* the arm of the long maze (in a $2d$ interval) to simulate the number of midline-crossings within the arm (short maze: $d + k$, long maze: $2d + k$) and adding the observed number of crossings in the intersection (k) as the overall simulated number of post cul-de-sac crossings in a trial. (D) Bootstrapping the (trimmed) $\#crossings$ made in the long maze preserves parity crossings tendencies in shorter mazes and replicates the observed $\#crossings$ distribution in the short maze. To consider the expected distributions of $\#crossings$ we counted, for each trial in the long maze, the number of first crossing attained within the interval $r \cdot d + k$, where $r = \{2, 1.9, 1.66, 1.5, 1.33, 1.1, 1, 0.9, 0.66, 0.5, 0.33, 0.1\}$ (D1-D12, respectively). We computed the expected distributions for each maze with arm length r by bootstrapping trials from the corresponding trimmed post cul-de-sac $\#crossings$ ($n_{samples} = 10{,}000$ and $n_{trials} = 7{,}360$ in each sample). Orange line and shaded area: expected $\#crossings$ pdf in the arm and intersection (a. & i.), averaged over 10,000 simulations and the simulations' std, respectively. Filled histogram in D1 & D7: observed $\#crossings$ pdf (as in B, C and Fig. 3D, bottom). D13: Fraction of even post cul-de-sac $\#crossings$ across all trials in the sample. Dashed: fraction of even $\#crossings$ observed in the short (black) and long (blue) mazes. Orange: average (line) and std (error bars) of the fraction of even $\#crossings$ across samples for each r. (E) Distributions of post cul-de-sac $\#crossings$ of individual flies in the long maze with largest (top) intermediate (center) and smallest (bottom) P_{Even} values (real data).

bioRxiv preprint doi: https://doi.org/10.1101/2024.09.04.611038; this version posted September 5, 2024. The copyright holder for this preprint (which was not certified by peer review) is the author/funder, who has granted bioRxiv a license to display the preprint in perpetuity. It is made available under aCC-BY 4.0 International license.

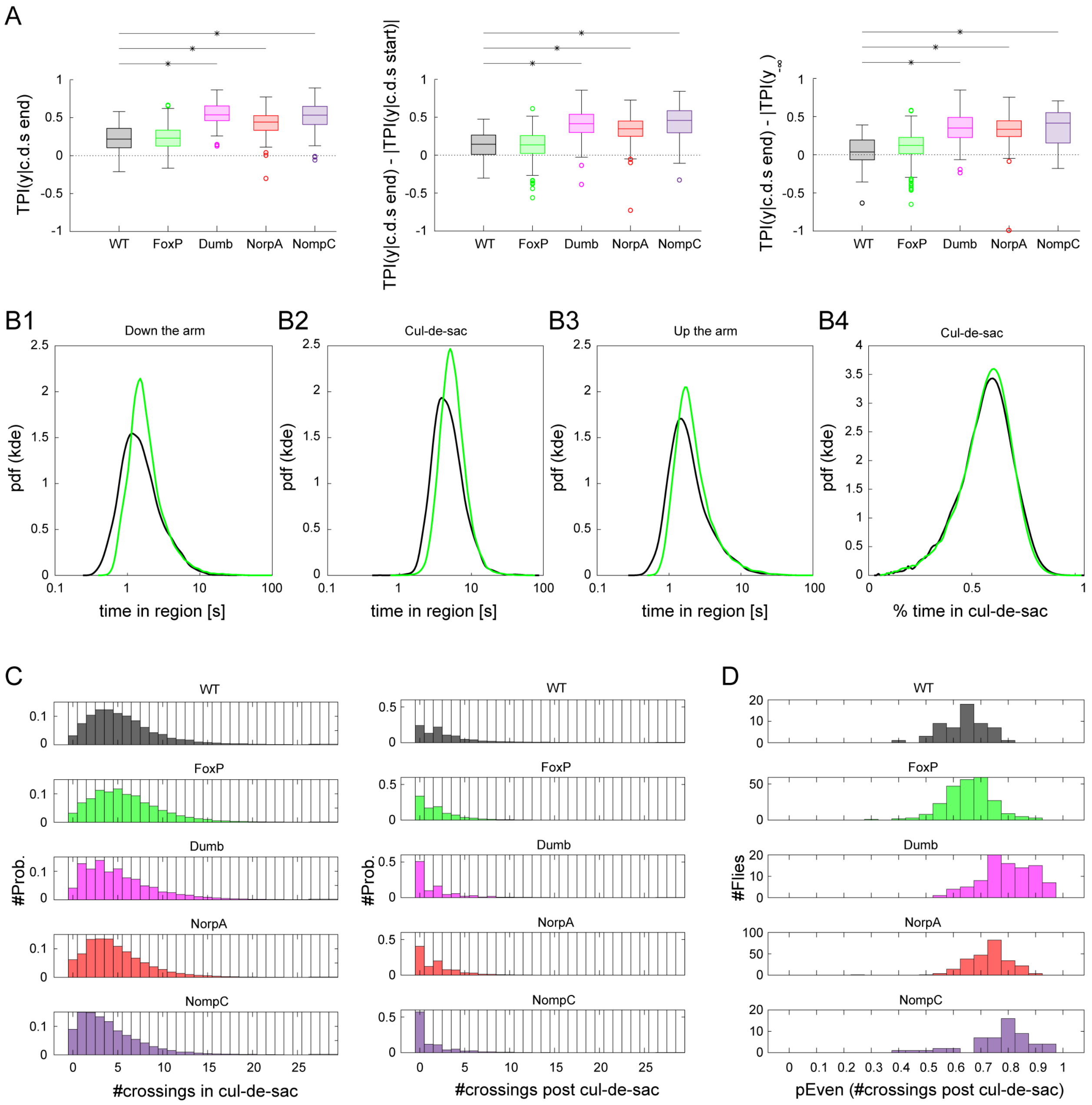

Figure S6. Mutants and WT comparisons. (A) TPI increase in the cul-de-sac across genetic lines: WT (black, $n = 55$), FoxP (green, $n = 243$), dumb (magenta, $n = 90$), NorpA (red, $n = 247$) and NompC (purple, $n = 54$). For each fly in each line, we computed the TPI value, $TPI(yRange)$, at the last location within the cul-de-sac ($yRange = \{0.2 \leq y \leq 0.3\}$, left), its difference with the TPI value at the last location within the cul-de-sac ($yRange = \{-0.3 \leq y \leq -0.2\}$, middle) and its difference with the left tail value of the $TPI(Y_{-\infty})$ (right). Each box plot depicts median (central mark), 25th and 75th percentiles (bottom and top edges of the box), most extreme data points not considered outliers (whiskers), and outliers (individual circles) across a genetic line. Asterisks depict significant differences between WT and mutant line (two-sided Wilcoxon rank sum test, corrected for multiple comparisons: $\alpha = 0.05/4$; left: $p_{WT,FoxP} = 0.67$, $p_{WT,dumb} < 0.001$,

bioRxiv preprint doi: https://doi.org/10.1101/2024.09.04.611038; this version posted September 5, 2024. The copyright holder for this preprint (which was not certified by peer review) is the author/funder, who has granted bioRxiv a license to display the preprint in perpetuity. It is made available under aCC-BY 4.0 International license.

$p_{WT,NorpA} < 0.001$, $p_{WT,NompC} < 0.001$, middle: $p_{WT,FoxP} = 0.87$, $p_{WT,dumb} < 0.001$, $p_{WT,NorpA} < 0.001$, $p_{WT,NompC} < 0.001$, right: $p_{WT,FoxP} = 0.07$, $p_{WT,dumb} < 0.001$, $p_{WT,NorpA} < 0.001$, $p_{WT,NompC} < 0.001$). (B) Probability density functions (PDFs) of the absolute (to not confuse with relative) time spent walking down the arm (B1, $-1 < y < -0.34$), within the cul-de-sac (B2, $-0.34 < y < 0.34$) and walking up the arm (right, $-0.34 < y < 1$) for WT (black) and FoxP (green) flies in the short. Each curve depicts a Kernel smoothing function estimate over log10 of the time spent in all trials across the sample. B4: Pdf of the fraction of time spent within the cul-de-sac (out of the total time spent between $-1 < y < 1$) across all trials in a sample, using a linear kernel smoothing function estimate. (C) Distributions of the number of midline-crossings within the cul-de-sac (left) and post cul-de-sac (right, $\#crossings$) across all trials made by flies in a genetic line (color-coded as A; $n_{trials,WT} = 7904$, $n_{trials,FoxP} = 34{,}764$, $n_{trials,dumb} = 12{,}391$, $n_{trials,NorpA} = 54{,}711$, $n_{trials,NompC} = 8{,}255$,). (D) Distributions of P_{Even}, the fraction of trials with even post-cul-de-sac $\#crossings$ across flies in each genetic line (colors and sample-sizes as A).

bioRxiv preprint doi: https://doi.org/10.1101/2024.09.04.611038; this version posted September 5, 2024. The copyright holder for this preprint (which was not certified by peer review) is the author/funder, who has granted bioRxiv a license to display the preprint in perpetuity. It is made available under aCC-BY 4.0 International license.

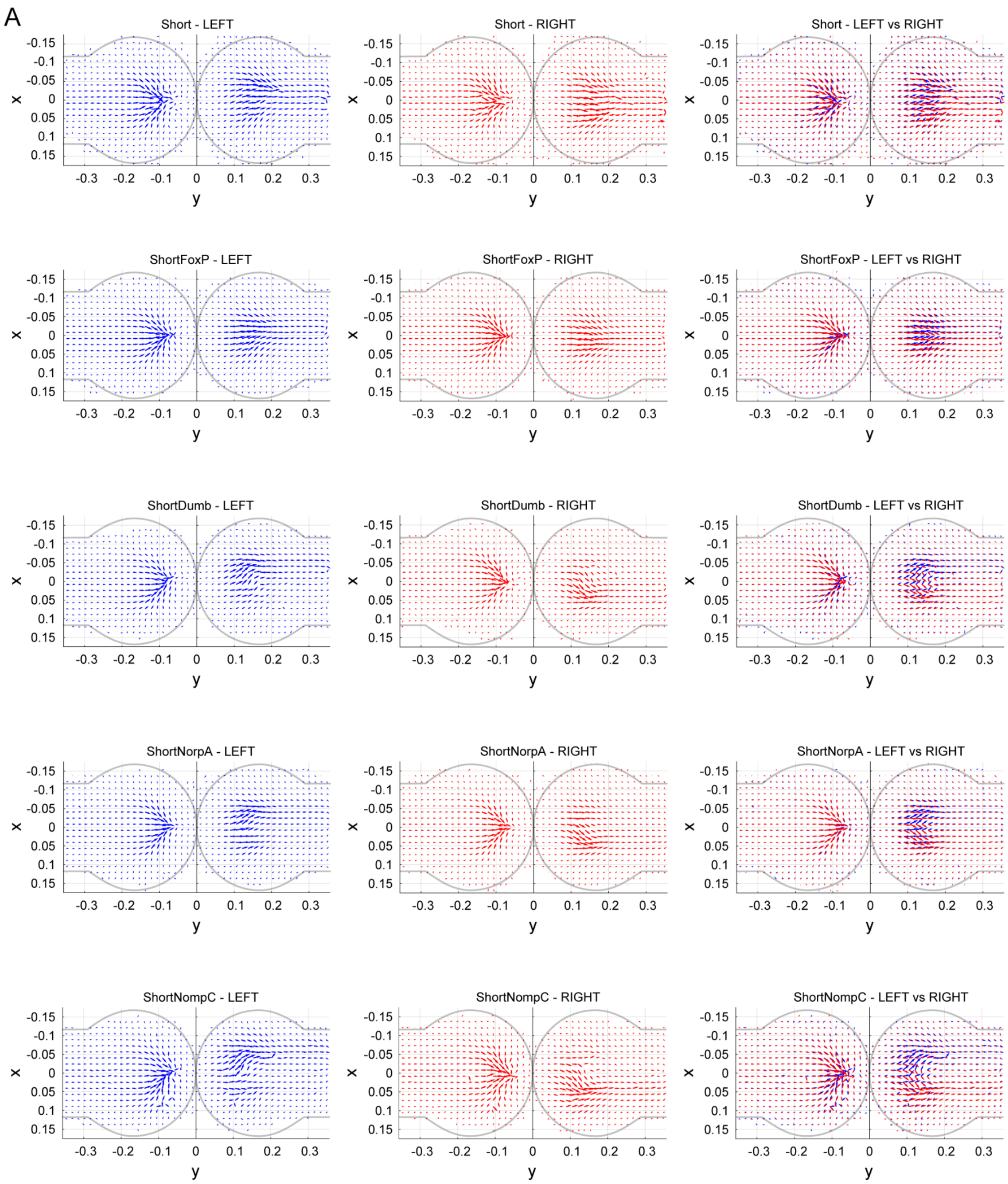

Figure S7. Quiver plots depicting average motion within the cul-de-sac across flies in each genetic line: WT $(n = 55)$, FoxP $(n = 243)$, dumb $(n = 90)$, NorpA $(n = 247)$ and NompC $(n = 54)$. Quivers are computed separately for inward $(y < 0)$ and outward $(y > 0)$ motions and separately for left turns (left panel, blue) and right turn (middle panel, red), based on flies' corresponding velocity vectors (Supporting Information). In each 2D bin, the vector direction

bioRxiv preprint doi: https://doi.org/10.1101/2024.09.04.611038; this version posted September 5, 2024. The copyright holder for this preprint (which was not certified by peer review) is the author/funder, who has granted bioRxiv a license to display the preprint in perpetuity. It is made available under aCC-BY 4.0 International license.

represents average motion direction across all flies, while the vector length indicates the relative frequency of visits to that bin (Supporting Information). Right panel: left and right turns, overlaid.

bioRxiv preprint doi: https://doi.org/10.1101/2024.09.04.611038; this version posted September 5, 2024. The copyright holder for this preprint (which was not certified by peer review) is the author/funder, who has granted bioRxiv a license to display the preprint in perpetuity. It is made available under aCC-BY 4.0 International license.

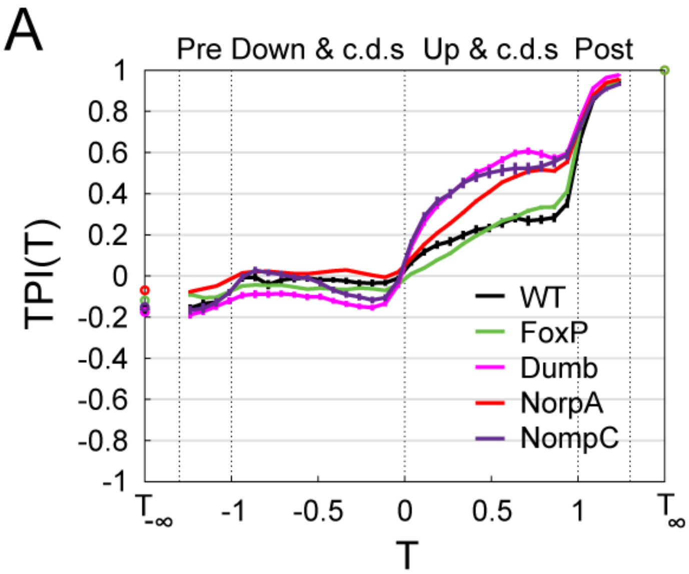

Figure S8. Average TPI curves in the temporal domain across genetic lines: WT (black, $n = 55$), FoxP (green, $n = 243$), dumb (magenta, $n = 90$), NorpA (red, $n = 247$) and NompC (purple, $n = 54$). Error bars denote SEM.

bioRxiv preprint doi: https://doi.org/10.1101/2024.09.04.611038; this version posted September 5, 2024. The copyright holder for this preprint (which was not certified by peer review) is the author/funder, who has granted bioRxiv a license to display the preprint in perpetuity. It is made available under aCC-BY 4.0 International license.

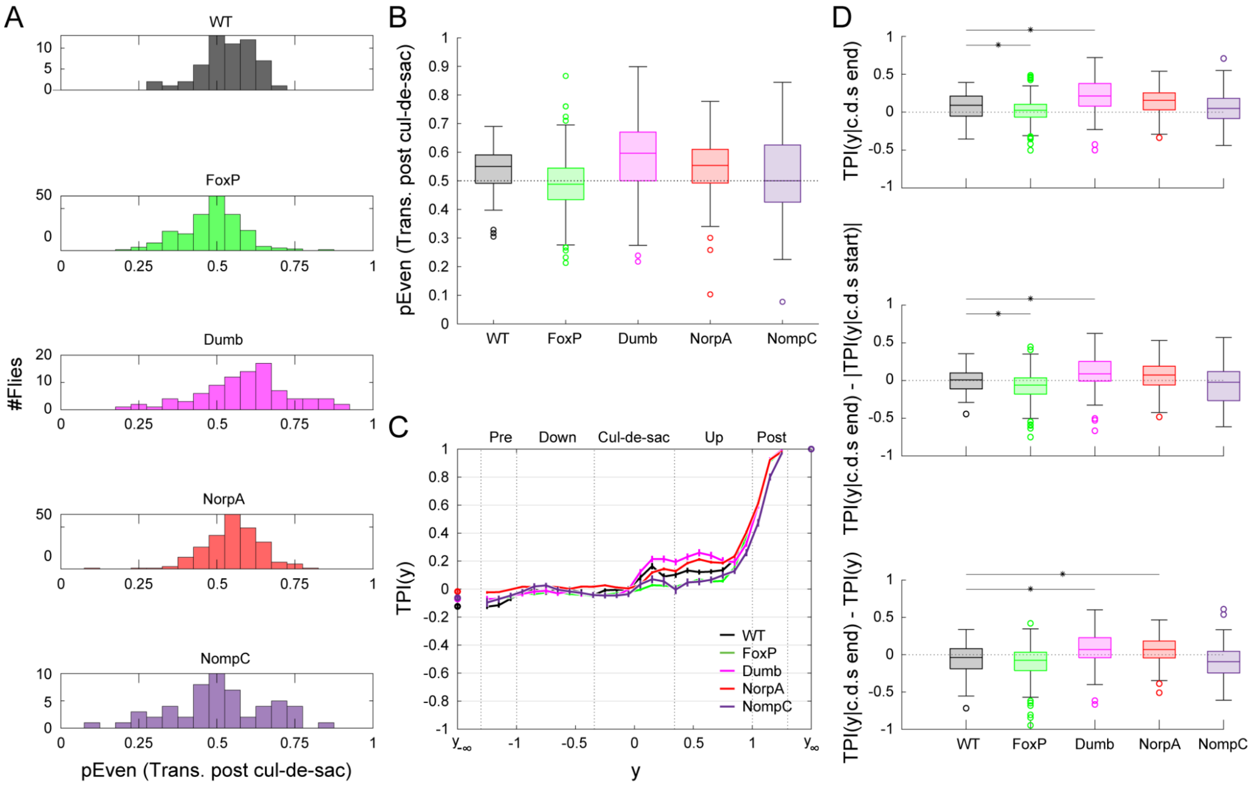

Figure S9. Exclusion of zero-crossings trials. (A) Distributions of the fractions of trials with even $\#crossings$ out of all trials with non-zero post cul-de-sac midline-crossing across flies in each genetic line: WT ($n = 55$, black), FoxP ($n = 243$, green), dumb ($n = 90$, magenta), NorpA ($n = 247$, red) and NompC ($n = 54$, purple). (B) Box plots summarizing the fractions in A across flies in each generic line, color-coded as in A. Each box depicts the median (central mark), 25th and 75th percentiles (bottom and top edges of the box), most extreme data points not considered outliers (whiskers), and outliers (individual circles) across a genetic line. Across all trials in a genetic line, the fractions of even, non-zero post cul-de-sac midline-crossing are: 0.5409 ± 0.0064, 0.4864 ± 0.0033, 0.6025 ± 0.0063, 0.5539 ± 0.0028, 0.5125 ± 0.0084 ($fraction \pm SE$) for the WT, FoxP, dumb, NorpA and NompC lines, respectively (see Fig. S10 for the fractions of trials with zero post cul-de-sac midline-crossings), and 0.5746 ± 0.0061 for WT flies in the long maze (not shown). (C) Average TPI curves in the spatial domain across genetic lines, after excluding trials with zero post cul-de-sac midline-crossings, color-codes and sample sizes as in A. Error bars denote SEM. (D) TPI increases in the cul-de-sac across genetic lines (as in 6A), after excluding trials with zero post cul-de-sac midline-crossings. For each fly in each line, we excluded all trial with zero post cul-de-sac midline-crossings and computed the TPI value over the remaining trials at the last location within the cul-de-sac ($yRange = \{0.2 \le y \le 0.3\}$, top), its difference with the TPI value at the last location within the cul-de-sac ($yRange = \{-0.3 \le y \le -0.2\}$, center) and its difference with the left tail value of the $TPI(Y_{-\infty})$ (bottom). Each box plot depicts median (central mark), 25th and 75th percentiles (bottom and top edges of the box), most extreme data points not considered outliers (whiskers), and outliers (individual circles) across a genetic line. Asterisks depict significant differences between WT and mutant line (two-sided Wilcoxon rank sum test, corrected for multiple comparisons: $\alpha = 0.05/4$; top: $p_{WT,FoxP} = 0.004$,

bioRxiv preprint doi: https://doi.org/10.1101/2024.09.04.611038; this version posted September 5, 2024. The copyright holder for this preprint (which was not certified by peer review) is the author/funder, who has granted bioRxiv a license to display the preprint in perpetuity. It is made available under aCC-BY 4.0 International license.

$p_{WT,dumb} < 0.001$, $p_{WT,NorpA} = 0.036$, $p_{WT,NompC} = 0.328$, center: $p_{WT,FoxP} = 0.002$, $p_{WT,dumb} = 0.005$, $p_{WT,NorpA} = 0.040$, $p_{WT,NompC} = 0.222$, bottom: $p_{WT,FoxP} = 0.079$, $p_{WT,dumb} < 0.001$, $p_{WT,NorpA} < 0.001$, $p_{WT,NompC} = 0.234$). Color-codes and sample sizes are as in A.

bioRxiv preprint doi: https://doi.org/10.1101/2024.09.04.611038; this version posted September 5, 2024. The copyright holder for this preprint (which was not certified by peer review) is the author/funder, who has granted bioRxiv a license to display the preprint in perpetuity. It is made available under aCC-BY 4.0 International license.

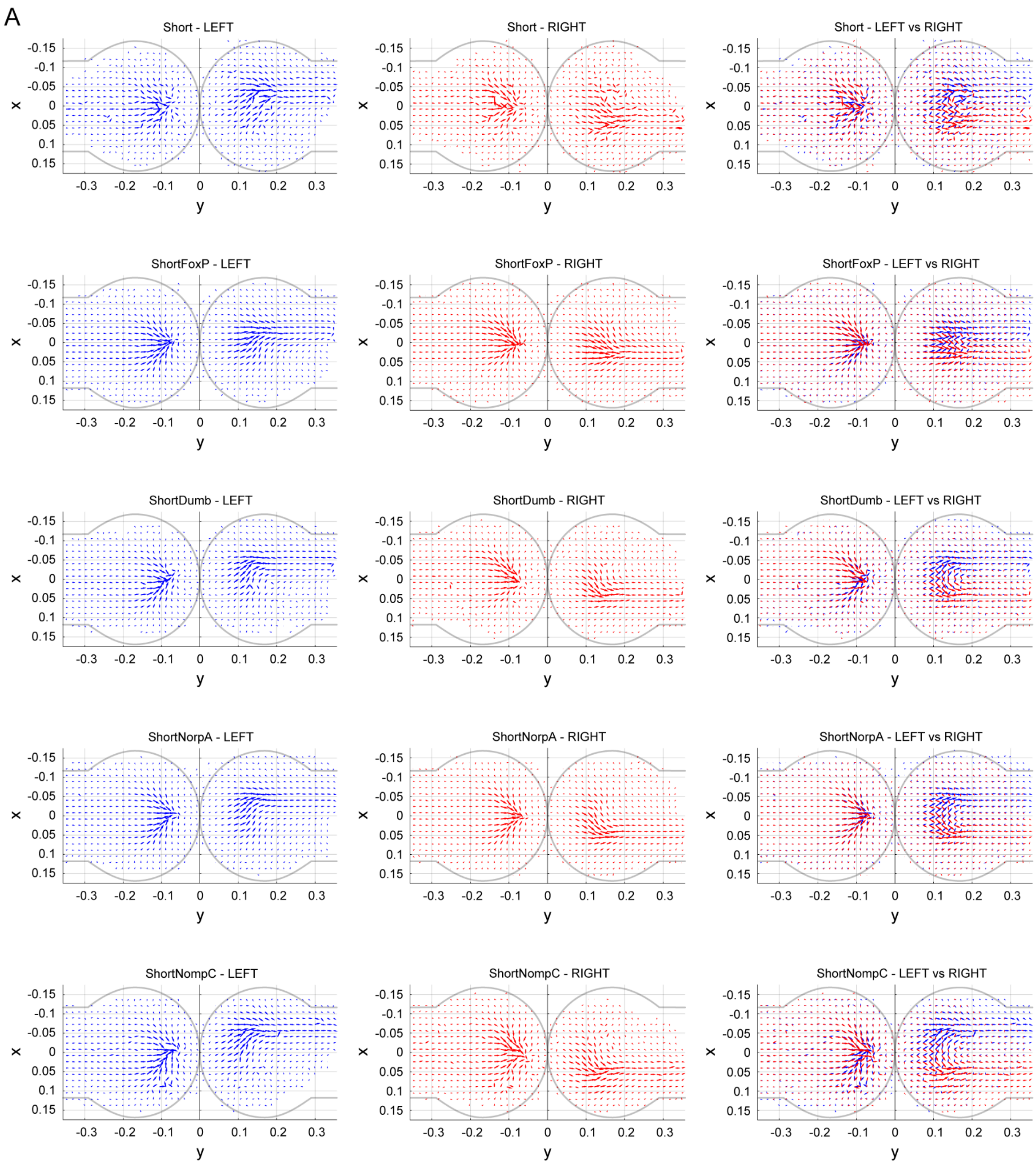

Figure S10. Zero-crossings quiver plots. Quiver plots as in S7, but including only trials in which there were no post cul-de-sac midline-crossings (trials in which $\#crossings = 0$; 24%, 34%, 51%, 24%, 40%, and 59% of the trials in the WT, FoxP, dumb, NorpA, and NompC lines, respectively).

bioRxiv preprint doi: https://doi.org/10.1101/2024.09.04.611038; this version posted September 5, 2024. The copyright holder for this preprint (which was not certified by peer review) is the author/funder, who has granted bioRxiv a license to display the preprint in perpetuity. It is made available under aCC-BY 4.0 International license.

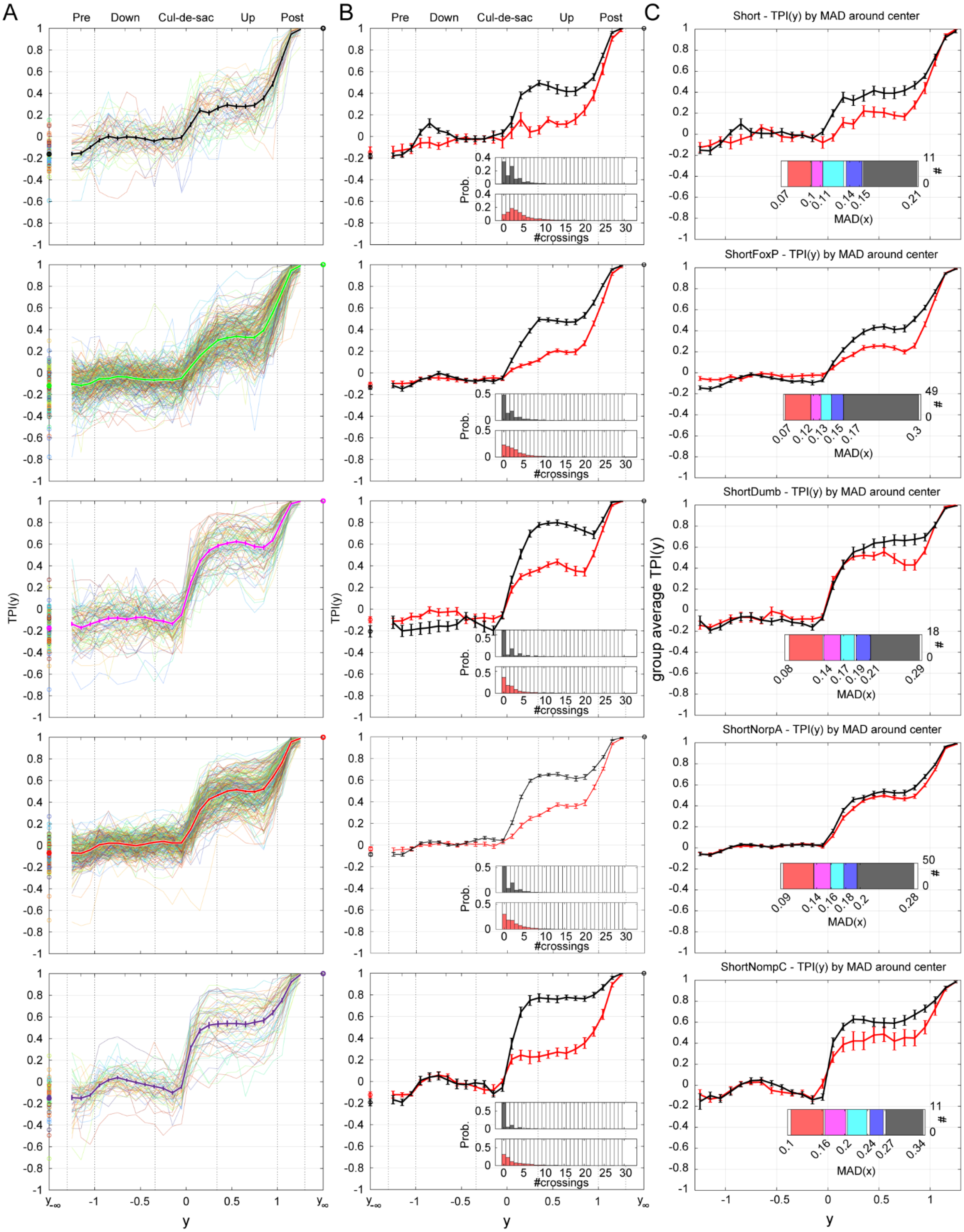

bioRxiv preprint doi: https://doi.org/10.1101/2024.09.04.611038; this version posted September 5, 2024. The copyright holder for this preprint (which was not certified by peer review) is the author/funder, who has granted bioRxiv a license to display the preprint in perpetuity. It is made available under aCC-BY 4.0 International license.

Figure S11. TPI of mutant and WT and their dependence on lateral tendencies. (A) Average TPI curve in the spatial domain across genetic lines (top to bottom: WT: black, $n = 55$; FoxP: green, $n = 243$; dumb: magenta, $n = 90$; NorpA: red, $n = 247$; NompC: purple, $n = 54$), overlaid on the TPI curves of individual flies. Error bars denote SEM. (B) Average TPIs for flies with lowest (red, 20% of the flies) and highest (black, 20% of the flies) P_{Even} values in each genetic line (computed for each genetic line as in Fig. 2D). Inset: Probability mass functions of $\#crossings$ in each group (color coded as in the main panel). (C) As in B, for global lateral tendencies: Average TPIs for flies with lowest and highest MAD values (red and black, respectively; 20% of the flies in each group; computed for each genetic line as in Fig. 2E). Inset: distribution of MAD scores over the sample.

bioRxiv preprint doi: https://doi.org/10.1101/2024.09.04.611038; this version posted September 5, 2024. The copyright holder for this preprint (which was not certified by peer review) is the author/funder, who has granted bioRxiv a license to display the preprint in perpetuity. It is made available under aCC-BY 4.0 International license.

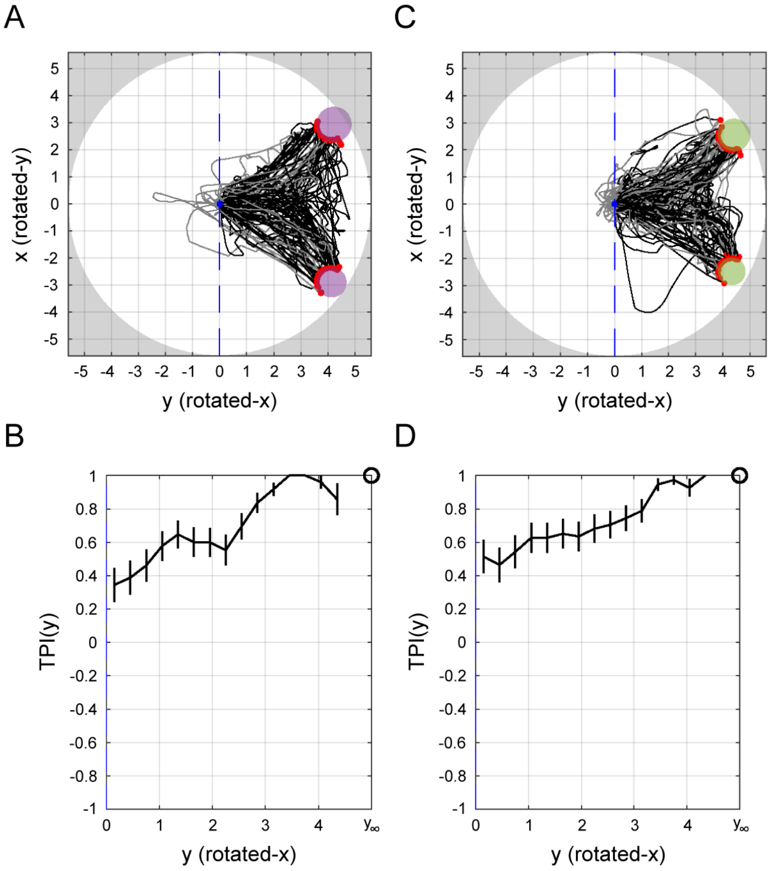

Figure S12. TPI of flies ($n = 30$) for the decision task in the study of Sridhar and colleagues (2021). Flies navigated a circular arena towards identical, equidistant targets. (A) Trajectories of all flies for targets (purple) that were approximately 70 degrees from the initial position of the fly ($n_{trials} = 111$). Black: trajectories with $min(y) > 0$ ($n_{trials} = 85$). Gray: trajectories with min(y)<0 ($n_{trials} = 26$). (B) TPI, computed over all the black trajectories in A ($n_{trials} = 111$). Error bars denote SE. (C) As in A, for targets (green) that were approximately 60 degrees from the initial position of the fly ($n_{trials} = 111$; black: $n_{trials} = 74$, gray: $n_{trials} = 37$). (D) As in B, for the black trajectories in C ($n_{trials} = 74$). Note that due to the small number of trials per fly, the TPI curves in B and D are computed over all trials in a condition.

bioRxiv preprint doi: https://doi.org/10.1101/2024.09.04.611038; this version posted September 5, 2024. The copyright holder for this preprint (which was not certified by peer review) is the author/funder, who has granted bioRxiv a license to display the preprint in perpetuity. It is made available under aCC-BY 4.0 International license.

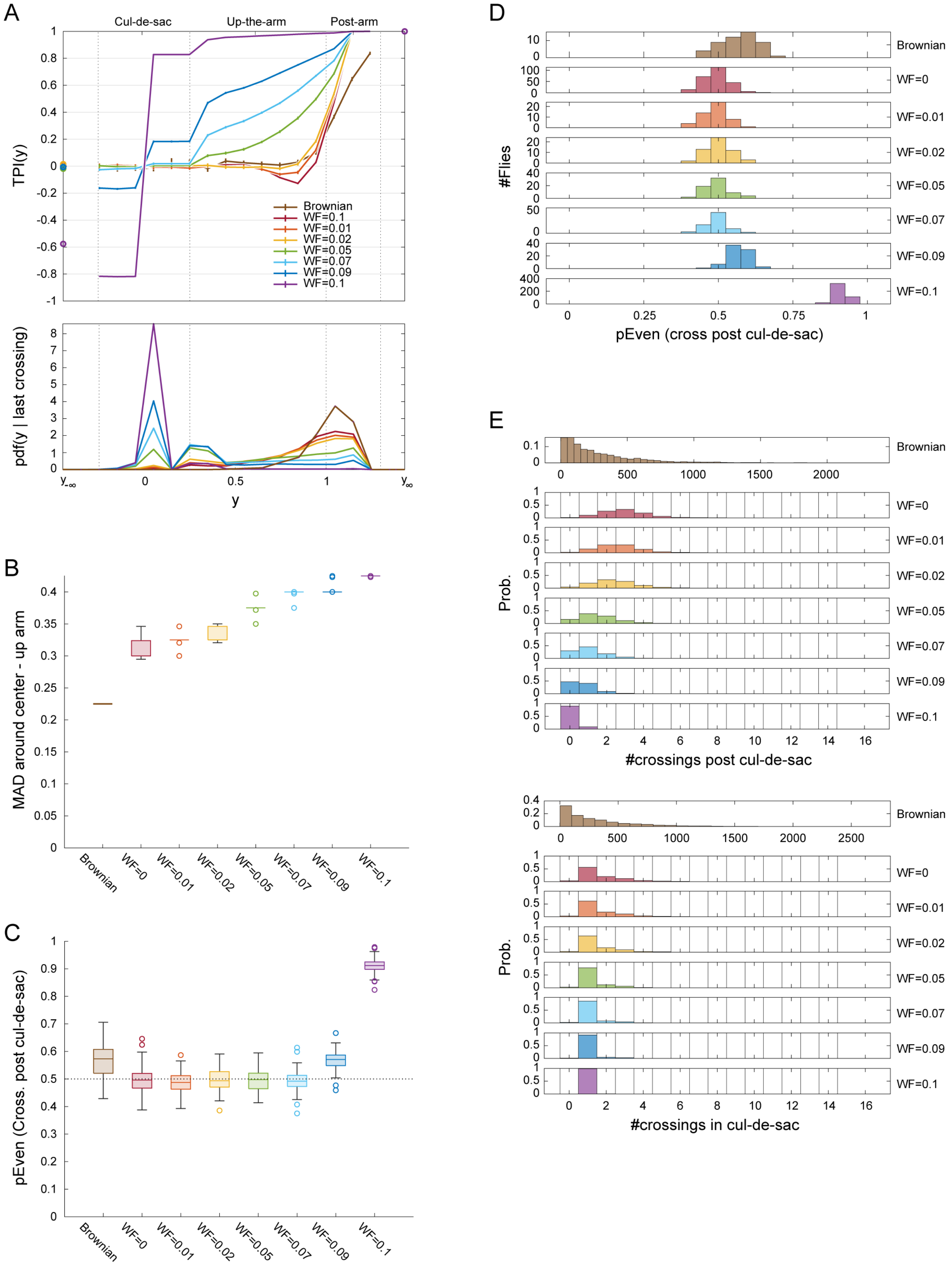

bioRxiv preprint doi: https://doi.org/10.1101/2024.09.04.611038; this version posted September 5, 2024. The copyright holder for this preprint (which was not certified by peer review) is the author/funder, who has granted bioRxiv a license to display the preprint in perpetuity. It is made available under aCC-BY 4.0 International license.

Figure S13. Agent based modeling (ABM). (A) Average TPI curves in the spatial domain and corresponding PDFs of last midline-crossings across AMBs: Brownian ABM (brown, $n = 50$), $WF = 0$ (red, $n = 251$), $WF = 0.01$ (orange, $n = 51$), $WF = 0.02$ (yellow, $n = 54$), $WF = 0.05$ (green, $n = 66$), $WF = 0.07$ (cyan, $n = 72$), $WF = 0.09$ (blue, $n = 78$), and $WF = 0.1$ (purple, $n = 457$). Top: Average TPI curve in the spatial domain of each ABM (note the narrow abscisa compared to Fig. 4A, top). Error bars denote SEM. Bottom: PDF of last midline-crossing (LMC), $PDF(y \mid LMC)$, across all trials and flies in each ABM. (B) Global lateral tendencies. Box plot depicting $MAD(x|0.34 < y < 1)$ scores (computed as in Fig. 4B, top) for each sample. For each box, the central mark indicates the median, bottom and top edges of the box indicate the 25th and 75th percentiles, whiskers extend to the most extreme data points not considered outliers, and outliers are plotted individually using symbols. (C) Local lateral tendencies. Box plot (as in B, bottom) depicting P_{Even} across each sample. (D) Distributions of P_{Even}, the fraction of trials with even $\#crossings$ across agents in each ABM. (E) Distributions of the number of midline-crossings post cul-de-sac (left, $\#crossings$) and within the cul-de-sac (right) across all trials made by agents in a given ABM (n_{trials} is 3656, 37458, 7840, 8426, 11957, 14185, 15680, 88564 for the Brownian ABM, $WF = 0$, $WF = 0.01$, $WF = 0.02$, $WF = 0.05$, $WF = 0.07$, $WF = 0.09$ and $WF = 0.1$, respectively).

bioRxiv preprint doi: https://doi.org/10.1101/2024.09.04.611038; this version posted September 5, 2024. The copyright holder for this preprint (which was not certified by peer review) is the author/funder, who has granted bioRxiv a license to display the preprint in perpetuity. It is made available under aCC-BY 4.0 International license.

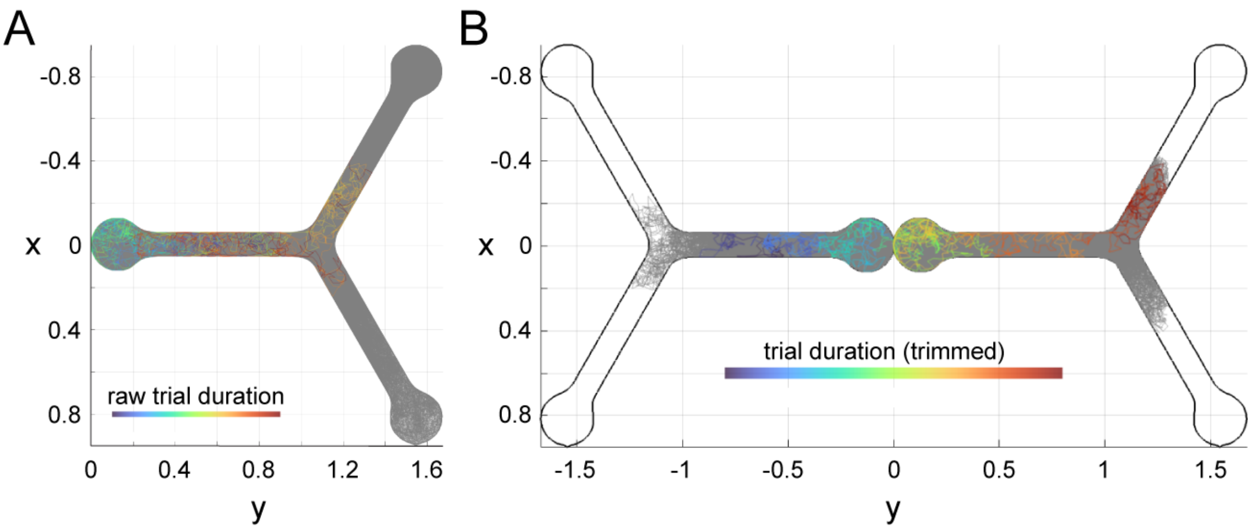

Figure S14. Trajectory trimming and correction for the Brownian ABM simulations. (A) Trajectory of an example trial for one simulated fly (color temperature denotes time progression), overlayed over multiple trajectories of its remaining bottom trials (gray). (B) Corrected and trimmed trajectory. The trajectory in (A) is trimmed such that it includes motion from the first arrival (from above) at the vertical center (y~0.65) of the bottom arm, until the first time $y = 1.3$ is reached from below. The trimmed trajectory is then corrected (as in Fig. 1B-C).

bioRxiv preprint doi: https://doi.org/10.1101/2024.09.04.611038; this version posted September 5, 2024. The copyright holder for this preprint (which was not certified by peer review) is the author/funder, who has granted bioRxiv a license to display the preprint in perpetuity. It is made available under aCC-BY 4.0 International license.

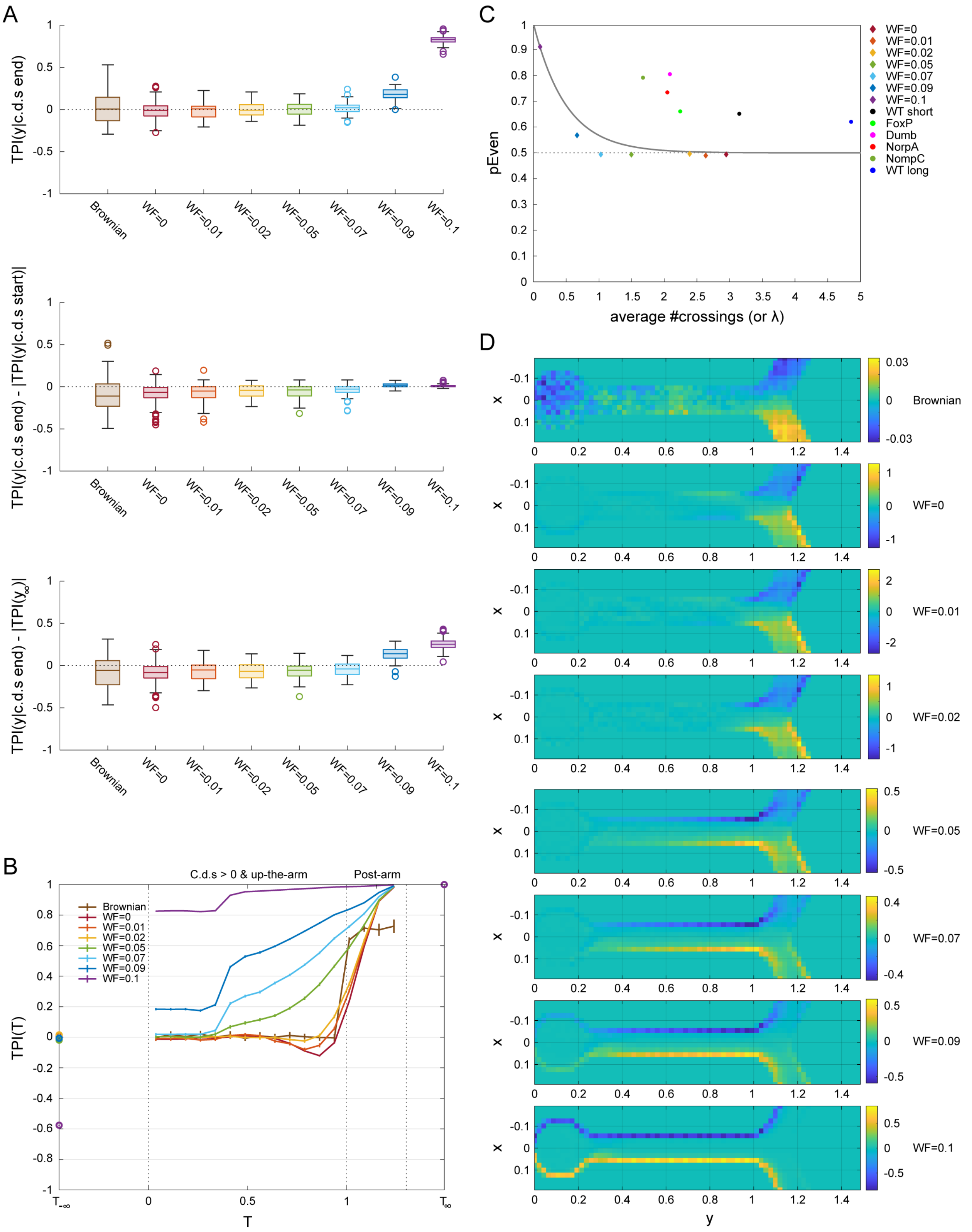

Figure S15. Agent based modeling (ABM). (A) TPI increase in the cul-de-sac across ABMs: Brownian ABM (brown, $n = 50$), $WF = 0$ (red, $n = 251$), $WF = 0.01$ (orange, $n = 51$), $WF = 0.02$ (yellow, $n = 54$), $WF = 0.05$ (green, $n = 66$), $WF = 0.07$ (cyan, $n = 72$), $WF = 0.09$ (blue, $n =$

bioRxiv preprint doi: https://doi.org/10.1101/2024.09.04.611038; this version posted September 5, 2024. The copyright holder for this preprint (which was not certified by peer review) is the author/funder, who has granted bioRxiv a license to display the preprint in perpetuity. It is made available under aCC-BY 4.0 International license.

78), and $WF = 0.1$ (purple, $n = 457$). Computed as in S6A. (B) Average TPI curves in the temporal domain across ABMs. Computed as in S8. Note the narrow abscisa compared to Fig. S8. (C) Poisson predictions *vs* observed parity tendencies. Symbols: observed P_{Even} as a function of the observed average $\#crossings$ across all trials in each of the genetic lines (diamonds) or ABMs (circles). Black curve: Poisson prediction. Expected $P_{Even}(\lambda)$ under the assumption that $\#crossings$ in a trial is Poisson distributed with λ (the expected value). Genetic lines' n_{trials} and ABMs' colors: 7904 black, 34764 green, 12391 magrenta, 54711 red, 8255 purple, and 7360 blue for WT, FoxP, dumb, NorpA, NompC, and WT in the long maze, respectively. ABM n_{trials} and colors: 3656 brown, 37458 red, 7840 orange, 8426 yellow, 11957 green, 14185 cyan, 15680 blue, and 88564 purple for the Brownian ABM, $WF = 0$, $WF = 0.01$, $WF = 0.02$, $WF = 0.05$, $WF = 0.07$, $WF = 0.09$ and $WF = 0.1$, respectively. (D) Heat maps (as in Fig. 2D, bottom) depicting the difference between the bivariate histograms of right and left turn-decisions for upward traversal ($y > 0$). Color temperature denotes probability (colorbar).
